# Laughter as a paradigm of socio-emotional signal processing in dementia

**DOI:** 10.1016/j.cortex.2021.05.020

**Published:** 2021-09

**Authors:** Harri Sivasathiaseelan, Charles R. Marshall, Elia Benhamou, Janneke E.P. van Leeuwen, Rebecca L. Bond, Lucy L. Russell, Caroline Greaves, Katrina M. Moore, Chris J.D. Hardy, Chris Frost, Jonathan D. Rohrer, Sophie K. Scott, Jason D. Warren

**Affiliations:** a Dementia Research Centre, UCL Queen Square Institute of Neurology, University College London, London, United Kingdom; b Preventive Neurology Unit, Wolfson Institute of Preventive Medicine, Queen Mary University of London, London, United Kingdom; c Department of Medical Statistics, Faculty of Epidemiology and Population Health, London School of Hygiene and Tropical Medicine, London, United Kingdom; d Institute of Cognitive Neuroscience, UCL Queen Square Institute of Neurology, University College London, London, United Kingdom

**Keywords:** Laughter, Vocal emotion, Authenticity, Social cognition, Frontotemporal dementia, Progressive aphasia, Alzheimer's disease, Numerophilia

## Abstract

Laughter is a fundamental communicative signal in our relations with other people and is used to convey a diverse repertoire of social and emotional information. It is therefore potentially a useful probe of impaired socio-emotional signal processing in neurodegenerative diseases. Here we investigated the cognitive and affective processing of laughter in forty-seven patients representing all major syndromes of frontotemporal dementia, a disease spectrum characterised by severe socio-emotional dysfunction (twenty-two with behavioural variant frontotemporal dementia, twelve with semantic variant primary progressive aphasia, thirteen with nonfluent-agrammatic variant primary progressive aphasia), in relation to fifteen patients with typical amnestic Alzheimer's disease and twenty healthy age-matched individuals. We assessed cognitive labelling (identification) and valence rating (affective evaluation) of samples of spontaneous (mirthful and hostile) and volitional (posed) laughter versus two auditory control conditions (a synthetic laughter-like stimulus and spoken numbers). Neuroanatomical associations of laughter processing were assessed using voxel-based morphometry of patients' brain MR images. While all dementia syndromes were associated with impaired identification of laughter subtypes relative to healthy controls, this was significantly more severe overall in frontotemporal dementia than in Alzheimer's disease and particularly in the behavioural and semantic variants, which also showed abnormal affective evaluation of laughter. Over the patient cohort, laughter identification accuracy was correlated with measures of daily-life socio-emotional functioning. Certain striking syndromic signatures emerged, including enhanced liking for hostile laughter in behavioural variant frontotemporal dementia, impaired processing of synthetic laughter in the nonfluent-agrammatic variant (consistent with a generic complex auditory perceptual deficit) and enhanced liking for numbers (‘numerophilia’) in the semantic variant. Across the patient cohort, overall laughter identification accuracy correlated with regional grey matter in a core network encompassing inferior frontal and cingulo-insular cortices; and more specific correlates of laughter identification accuracy were delineated in cortical regions mediating affective disambiguation (identification of hostile and posed laughter in orbitofrontal cortex) and authenticity (social intent) decoding (identification of mirthful and posed laughter in anteromedial prefrontal cortex) (all *p* < .05 after correction for multiple voxel-wise comparisons over the whole brain). These findings reveal a rich diversity of cognitive and affective laughter phenotypes in canonical dementia syndromes and suggest that laughter is an informative probe of neural mechanisms underpinning socio-emotional dysfunction in neurodegenerative disease.

## 1 Introduction

Alterations in emotional and social behaviour are prominent clinical features in a number of dementias, leading to significant distress and care burden (Galvin et al., 2017; Kandiah et al., 2016; Mioshi et al., 2013). The paradigmatic disorders of socio-emotional behaviour are the frontotemporal dementias (FTD), comprising three canonical clinical syndromes: behavioural variant frontotemporal dementia (bvFTD), semantic variant primary progressive aphasia (svPPA) and non-fluent/agrammatic primary progressive aphasia (nfvPPA). Whilst deficits in emotion recognition, empathy and social understanding are defining features of bvFTD (Rascovsky et al., 2011), they are also well documented in svPPA and nfvPPA (Hazelton et al., 2017; Irish et al., 2013; Midorikawa et al., 2017; Rohrer & Warren, 2010). Changes in social and emotional cognition are also increasingly recognised in Alzheimer's disease (AD) (Martinez et al., 2018). However, despite their significant impact, these changes are poorly understood and challenging to assess objectively. This is attributable both to the inherently complex and multifaceted nature of emotional and social behaviour and a lack of tractable models and instruments with which to measure these phenomena.

To date, studies of emotional and social signal processing in dementia have focussed largely on recognition and categorisation of facial expressions, characterising impairments across the FTD spectrum that particularly impact recognition of negative expressions and interpreting the expressions of other people (Rosen et al., 2004; Hutchings et al., 2017; Marshall et al., 2019; Bertoux et al., 2016; Caminiti et al., 2015; Downey et al., 2013). However, socio-emotional deficits in FTD extend to other sensory channels, in particular auditory signals (Snowden et al., 2008; Keane et al., 2002; Hsieh et al., 2013; Omar et al., 2011). Vocal paralinguistic affective signalling amplifies, contextualises or may even override verbal messages [as exemplified in affective prosody and sarcasm (Rankin et al., 2009; Voyer et al., 2016; Sascha et al., 2019; Agustus et al., 2018)]. Processing of such signals is impaired in bvFTD and PPA syndromes (Downey et al., 2015; Kipps et al., 2009; Marshall et al., 2018; Rankin et al., 2009; Rohrer et al., 2012). Impaired processing of emotional prosody has also been described in typical AD; here (as in nfvPPA) perhaps reflecting a more elementary deficit of auditory pattern analysis (Agustus et al., 2018; Horley et al., 2010; Rohrer et al., 2012; Testa et al., 2001). Nonverbal emotional vocalisations represent another essential component of social communication, enabling emotional signals to be broadcast rapidly even under conditions that would hinder visual signalling.

Among the cardinal nonverbal vocalisations that we use as humans, arguably the richest, most universal and most socially resonant is laughter. Laughter is phylogenetically ancient (Provine, 2017); in primates it serves to signal positive affect and affiliation, primarily during play and social grooming (Provine, 2013). It develops in human infants before speech (Scheiner et al., 2006) and is trans-cultural and socially ubiquitous (Sauter and Eisner 2013; Sauter et al., 2015). However, we seldom laugh alone, and laughter is extensively modulated by social context (Meyer et al., 2007; Scott et al., 2014; Szameitat et al., 2009a; Wildgruber et al., 2013): besides conveying mirth or conviviality, laughter may be used to taunt an opponent, express delight in another's misfortune (schadenfreude) or cover embarrassment. Even more frequently, laughter is voluntarily generated, or ‘posed’: unlike spontaneous laughter that is stimulus driven and emotionally tuned, voluntary laughter is not necessarily associated with any strong emotional experience but may rather facilitate affiliation or polite agreement (Scott et al., 2014). These laughter types are distinguished by their acoustic signatures (Szameitat et al., 2009b, 2011) and under experimental conditions, healthy subjects can reliably classify laughter even when non-auditory cues are removed (Szameitat et al., 2009a). However, because laughter can express wide variation in affect and authenticity within the frame of a single acoustic carrier, it is an intrinsically ambiguous stimulus: a characteristic that is mirrored in the multi-dimensionality of natural social scenarios more generally. Not surprisingly, the neural apparatus responsible for decoding and evaluating such a complex signal is elaborate. fMRI studies of laughter processing in the healthy brain have implicated distributed cerebral networks, encompassing superior temporal and inferior frontal cortices engaged in decoding auditory sequences, mesial temporal and insular regions mediating sensory-affective integration and emotional reactivity, and anteromedial prefrontal and orbitofrontal circuitry that appraises and evaluates affective sensory signals (Craig, 2009; Frühholz et al., 2016; Frühholz & Grandjean, 2013; Lavan et al., 2017; McGettigan et al., 2015; Wildgruber et al., 2013). Together these neural networks reconstitute much of the recently defined human social brain connectome (Alcalá-López et al., 2017).

As a neuropsychological tool, laughter is well equipped to expose subtle degrees of socio-emotional dysfunction in people with FTD, who typically struggle to resolve ambiguity and context in social situations, even while still performing relatively well on standard neuropsychological tests of emotion recognition (Clark, Nicholas et al., 2017; Downey, Mahoney et al., 2015; Snowden, Austin et al., 2008). Moreover, the neural substrates of laughter processing in the healthy brain are affected early and prominently in the course of major dementias, particularly FTD (Rohrer, 2012; Sivasathiaseelan et al., 2019), suggesting that laughter may constitute a neuroanatomically pertinent probe of socio-emotional processing in dementia. Indeed, neurodegenerative diseases have been linked to abnormalities of laughter behaviour in daily life. In the context of punctuating conversation, patients with bvFTD (and also AD) laugh less whereas patients with nfvPPA may laugh more than their healthy caregivers (Pressman et al., 2017); while patients with bvFTD and svPPA often laugh inappropriately, for example in response to others’ misfortune (Clark et al., 2016). However, processing of laughter has not been studied in detail in neurodegenerative disease.

Here we present the first investigation of the cognitive and affective processing of laughter in patients representing canonical FTD syndromes and AD, referenced to a group of healthy older individuals. We created a novel battery of stimuli, representing genuine mirthful and hostile laughter along with posed (volitional) laughter together with a synthetic, perceptually complex laughter-like stimulus. These stimulus conditions represent the various previously recognised categories of laughter subtypes and reflect the diverse socio-emotional signals that laughter can communicate. Whilst not mutually exclusive, these laughter signals can be categorised based on the sender's intent and the listener's reaction (McGettigan et al., 2015; Szameitat et al., 2009a; Wildgruber et al., 2013) and have been shown to have separable acoustic signatures (Lavan et al., 2016; Szameitat et al., 2009b, 2011) and to engage differentiated neural mechanisms (Lavan et al., 2017; McGettigan et al., 2015; Szameitat et al., 2010). The stimuli we employed here were designed to allow us to separately assess cognitive deficits ranging from primary perceptual (laughter-like signals *vs* natural laughter) through semantic emotional categorisations (mirthful *vs* hostile laughter) to social cognitive categorisation (posed *vs* spontaneous laughter). We assessed explicit identification (perceptual cognitive categorisation) of the laughter subtypes represented, alongside affective evaluation (valence rating of laughter subtypes) and in relation to daily life measures of socio-emotional reactivity. Neuroanatomical associations of laughter identification in the patient cohort were assessed using voxel-based morphometry.

Based on available evidence (Agustus et al., 2018; Clark et al., 2016; Downey et al., 2015; Fletcher et al., 2015b; Kipps et al., 2009; Pressman et al., 2017; Rankin et al., 2009; Rohrer et al., 2012), we hypothesised that impairments of laughter processing would be widespread across FTD and AD but would show dissociated patterns of deficits in different syndromes. We predicted more severe deficits in FTD syndromes than in AD, with a more elementary deficit of perceptual analysis in nfvPPA and more severe social and emotional processing deficits in svPPA and bvFTD. We further hypothesised that laughter identification in these diseases would have neuroanatomical correlates in distributed cerebral networks previously implicated in laughter processing in the healthy socio-emotional brain (Alcalá-López et al., 2017; Frühholz et al., 2016; Frühholz & Grandjean, 2013; Lavan et al., 2017; McGettigan et al., 2015; Szameitat et al., 2010), with partially separable correlates for different laughter subtypes and hub zones for signal salience, affective and mental state decoding in insula, orbitofrontal and medial prefrontal cortices, respectively.

## 2 Materials and methods

### 2.1 Participants

Forty-seven patients with a syndrome of FTD (22 with bvFTD, 12 with svPPA, 13 with nfvPPA) and 15 patients with typical amnestic AD were recruited from a specialist cognitive disorders clinic. All patients fulfilled consensus criteria for the relevant syndromic diagnosis (Dubois et al., 2014; Gorno-Tempini et al., 2011; Rascovsky et al., 2011), of mild to moderate severity. Twenty healthy older individuals with no history of neurological or psychiatric illness also participated. No participant had a history of significant hearing loss; peripheral hearing function was assessed using pure tone audiometry following a previously described procedure (Hardy et al., 2019) (details in Supplementary Material online) and composite hearing scores were included as covariates in behavioural, physiological and anatomical analyses. General neuropsychological assessment and brain MRI corroborated the syndromic diagnosis in all patients; no participant had radiological evidence of significant cerebrovascular damage.

To assess the relations between laughter processing and impairments of daily life emotional and social behaviour, the Modified Interpersonal Reactivity Index (mIRI) (Davis, 1983) and Revised Self-Monitoring Scale (RSMS) (Lennox & Wolfe, 1984) were completed by primary caregivers of patients with FTD syndromes. Whilst there is no standardised measure of social and emotional behaviour in dementia, the mIRI is a validated, widely used measure of cognitive and emotional empathy that has been administered previously to people with dementia (Eslinger et al., 2011) whilst the RSMS is a measure of sensitivity and responsiveness to others’ emotional expressions and behaviour that has been used in previous studies of both healthy and clinical populations (Shdo et al., 2018; Toller et al., 2018).

The study was approved by the University College London institutional ethics committee and all participants gave informed consent in accordance with the Declaration of Helsinki.

We report how we determined our sample size, all data exclusions, all inclusion/exclusion criteria, whether inclusion/exclusion criteria were established prior to data analysis, all manipulations, and all measures in the study.

The conditions of our ethics approval do not permit public archiving of anonymised study data. Readers seeking access to the data should contact the corresponding author; access will be granted to named individuals in accordance with ethical procedures governing the reuse of clinical data, including completion of a formal data sharing agreement and approval of the local ethics committee.

Legal copyright restrictions prevent public archiving of the various tests and assessment batteries used in this study, which can be obtained from the copyright holders in the cited references.

### 2.2 Creation of experimental stimuli

We created sound stimuli to represent each of the three major natural laughter categories of interest: mirthful (spontaneously reactive, involuntary laughter induced by an intrinsically amusing situation), hostile (spontaneous laughter in response to others’ misfortune or discomfiture, with the effect of taunting or deriding them) and posed (laughter produced volitionally with a more intentional, communicative purpose, generally in response to social cues and disproportionate to any felt amusement). Short samples of mirthful and posed laughter were derived from a previously published battery (McGettigan et al., 2015); additional examples of mirthful, posed and hostile laughter were derived from video clips publicly available on youtube.com. Highly identifiable examples of each laughter condition were selected based on an initial pilot experiment in healthy young adults.

In addition to these natural laughter conditions, we created two control stimulus conditions to allow us to interpret the affective response elicited by laughter stimuli. The first control condition was intended to calibrate for the effect of hearing a human voice, by establishing a baseline vocal condition that did not express any clear emotion: trials in this condition each comprised one of two male voices reading aloud a three-digit number with neutral intonation (this verbal carrier was chosen because nonverbal vocalisations that are not intended to convey emotion – e.g., yawning – have been shown in unpublished data from our laboratory to have affective connotations). The second control condition was intended to calibrate for the effect of hearing an affectively arousing, laughter-like signal: trials in this condition comprised samples of spectrally inverted laughter, synthesised digitally from raw recordings representing each of the laughter conditions using a previously described algorithm (Goll et al., 2010) (this stimulus retains the spectrotemporal complexity of laughter but is normally perceived as ‘alien’ and aversive (see Supplementary Material online). Stimuli in each of the control conditions were edited digitally to have the same general acoustic parameters as the laughter stimuli.

The final set of 80 stimuli used in the main experiment comprised 16 highly identifiable examples of each laughter condition plus 16 examples of each control condition. Further details of the stimulus set and examples of each condition are available in Supplementary Material online. Legal copyright restrictions prevent public archiving of the experimental stimuli used in this study. The stimuli will be made available unconditionally on request to the corresponding author.

### 2.3 Experimental paradigm

Participants were first familiarised with the experimental set-up and practice trials were delivered (using stimuli not subsequently used in the experiment proper) to ensure they understood the procedure and were able to comply. All auditory stimuli were delivered in randomised order at an individually comfortable, fixed listening level (approximately 70 dB) via AudioTechnica® ATH-M50X headphones from a notebook computer running Eyelink Experiment Builder software (SR Research, Ottawa, Canada) – this commercial software requires a license and does not produce any source code available for sharing.

In a first experimental session, all stimulus conditions were presented and the task on completion of each sound was to rate its valence on a modified 5-point Likert scale (1, very unpleasant; 5, very pleasant). In a second, separate experimental session, the laughter conditions were presented and the task on each trial was to decide if the sound represented mirthful (‘happy’), hostile (‘nasty’), posed (‘faked’) or spectrally inverted (‘computer’) laughter (the spoken number condition was not presented during this session). The separation of sessions was intended both to avoid the cognitive demands of dual tasks administered in a single session and to minimise any mutual priming between affective rating and identification of laughter conditions. No feedback about performance was given and no time limits were imposed.

### 2.4 Statistical analysis of general phenotypic and experimental behavioural data

Data were analysed using Stata14® (StataCorp, College Station, TX, USA). Between-group comparisons of continuous demographic and neuropsychological data were performed using analysis of variance (ANOVA) whilst analogous comparisons for categorical data (e.g., gender, handedness) were carried out using chi-squared tests.

Sound classification was a multiple-choice task and therefore unbiased hit rates (*Hu*) were computed for each laughter condition, to yield a measure of perceptual sensitivity taking into account both the hit rate and false alarm rate. The Hu measure was devised for use in category judgement experiments (Wagner, 1993), calculated as: *Hu* = (*Ai*/*Bi*) × (*Ai*/*Ci*), where *Ai* = frequency of hits, *Bi* = number of trials where *i* is target and *Ci* = frequency of *i* responses (hits and false alarms). This was converted to a percentage with a score of zero denoting chance performance.

These unbiased hit rates were compared amongst groups and conditions using a linear regression model, with diagnostic group, condition and their interactions, along with age, WASI Matrices score and composite audiometry score as predictor variables. Age has been recognised to impact emotion recognition in different modalities, including facial expressions (Gonçalves et al., 2018) and voice (Amorim et al., 2021; Chen, 2018). In the absence of any widely accepted, satisfactory standard measure of disease severity across dementia syndromes, we used WASI Matrices score as a covariate to adjust for the overall severity of cognitive dysfunction here. This measure indexes relevant cognitive processes (including abstract nonverbal reasoning and executive decision making) that are affected in the dementia syndromes under study here and might impact generically on performance in our laughter identification task. Importantly, WASI Matrices score places no demands on linguistic processing and is therefore not confounded by language decline in progressive aphasia syndromes. The non-independence of the repeated responses (across conditions) was accounted for by using robust (Huber-White) standard errors (Huber, 1967; White, 1980) that allowed for correlated responses by participant for construction of confidence intervals and hypothesis tests.

Bonferroni-corrected post hoc *t*-tests were carried out where a joint test of the group or condition effects or their interaction was statistically significant. So, for example, when making pairwise comparisons between five groups for a particular condition, the *p*-values were multiplied by ten. Similar modifications were made to 95% confidence intervals. An analogous approach to that for unbiased hit rates was used to compare valence judgements by group and condition.

For laughter identification, numbers of each type of error (out of 16) were analysed to look for any evidence of systematic bias or difference between groups. Separate logistic regression models were fitted for each type of error. These models included age, WASI matrices score and composite audiometry score as well as group as predictor variables. Since the distribution of the number of errors might not be binomial, robust Huber-White standard errors were used as above. In cases where the omnibus test of comparisons amongst groups was statistically significant, pairwise comparisons were made with Bonferroni adjustment for multiple comparisons as above.

Associations between total laughter identification accuracy and the two questionnaire-based measures of socio-emotional behaviour (mIRI and RSMS) as well as the British Picture Vocabulary Scale (BPVS; an index of general semantic competence) were assessed using a regression model incorporating age, audiometry score and WASI Matrices score as nuisance variables.

### 2.5 Brain image acquisition and analysis

Each patient had a sagittal 3-D magnetisation-prepared rapid-gradient-echo T1-weighted volumetric brain MR sequence (echo time/repetition time/inversion time 2.9/2200/900 msec, dimensions 256 256 208, voxel size 1.1 × 1.1 × 1.1 mm), acquired on a Siemens Prisma 3 T MRI scanner using a 32-channel phased-array head-coil. Pre-processing of brain images was performed using the New Segment and DARTEL toolboxes of SPM12 (www.fil.ion.ucl.ac.uk.spm), following an optimised protocol (Ridgway et al., 2008). Normalisation, segmentation and modulation of grey and white matter images were performed using default parameter settings and grey matter images were smoothed using a 6 mm full-width-at-half-maximum Gaussian kernel. A study-specific template mean brain image was created by warping all bias-corrected native space brain images to the final DARTEL template and calculating the average of the warped brain images. Total intracranial volume was calculated for each patient by summing grey matter, white matter and cerebrospinal fluid volumes after segmentation of tissue classes.

Following quality control of the pre-processed brain images, scans from 60 patients (13 AD, 22 bvFTD, 13 svPPA and 12 nfvPPA) were entered into the VBM analysis. A regression model was used to assess associations of regional grey matter volume (indexed as voxel intensity) with overall laughter identification score (percentage of all laughter trials accurately identified) for the combined patient cohort. In addition, grey matter associations with unbiased hit rates for each laughter condition were assessed in separate models across the combined patient cohort. Age, total intracranial volume and WASI Matrices score (included as a proxy for disease severity, to reduce variance attributable to advancing disease with widespread grey matter atrophy) were incorporated as covariates of no interest in all models. Statistical parametric maps of regional grey matter associations were assessed at threshold *p* < .05 after family-wise error (FWE) correction for multiple voxel-wise comparisons over the whole brain.

## 3 Results

### 3.1 General characteristics of participant groups

Demographic, clinical and neuropsychological characteristics of the participant groups are summarised in Table 1. Participant groups did not differ significantly in mean age [F(4,77) = 2.24, *p* = .07], gender distribution [χ^2^(4) = 2.02, *p* = .73], years of education [F(4,77) = 1.12, *p* = .35] or composite audiometry score [F(4,77) = 2.09, *p* = .09]. Mean duration of symptoms was not significantly different between the patient groups [F(3,58) = 2.19, *p* = .10]. General neuropsychological profiles were consistent with the syndromic diagnosis in each patient group.

**Table 1:** Demographic, clinical and general neuropsychological characteristics of participant groups.

Characteristic	Controls	AD	nfvPPA	svPPA	bvFTD
**Demographic**					
No. (male:female)	12:8	8:7	7:6	7:5	16:6
Age (years)	65.3 (6.3)	70.8 (6.2)	69.3 (10.0)	66.8 (7.2)	66.5 (6.2)
Handedness (right:left)	19:1	13:2	12:1	12:0	21:1
Education (years)	14.8 (3.1)	14.1 (2.5)	12.9 (2.4)	13.4 (2.5)	14.3 (3.0)
MMSE (/30)	29.5 (.9)	**20.1 (5.9)**	**22.1 (7.2)**	**21.6 (7.2)**	**24.8 (4.8)**
Symptom duration (years)	N/A	6.9 (2.9)	4.9 (1.3)	7.1 (2.4)	7.0 (3.0)
Genetic mutations (no.)	N/A	0	2 *GRN*	0	4 *MAPT* 3 C9orf72 2 *GRN*
No. taking donepezil	0	10	0	0	0
No. taking antidepressants	0	7	4	4	7
Audiometry score	26.3 (5.1)	30.7 (6.8)	29.5 (6.8)	27.7 (6.1)	32.2 (7.7)
**General neuropsychological**					
*Episodic memory*					
RMT words (/50)	48.3 (2.0)	**30.4 (6.0)**	**34.0 (8.9)**	**33.3 (7.8)**	**36.4 (8.7)**
RMT faces (/50)	42.5 (5.3)	**31.2 (7.8)**	**34.9 (6.9)**	**29.3 (2.8)**	**34.2 (8.1)**
*Executive skills*					
WASI Matrices (/32)	25.7 (4.2)	**11.9 (6.1)**^c^	**15.1 (9.5)**^c^	25.8 (4.7)	**18.2 (8.8)**^c^
D-KEFS Stroop:					
colour naming (s)	29.6 (5.5)	**58.9 (18.4)**	**76.1 (17.7)**	**51.6 (22.5)**^b^	**44.7 (18.0)**^b^
word reading (s)	22.1 (4.6)	**46.7 (24.4)**^b^	**65.5 (24.2)**	34.7 (12.2)^b^	29.5 (15.2)^b^
interference (s)	57.3 (17.8)	**139.3 (42.5)**	**151.5 (44.6)**	**99.4 (44.5)**^a^^,^^b^	**93.8 (44.4)**^a^^,^^b^
Trails A (s)	27.1 (6.1)	**98.7 (42.1)**	**77.3 (52.7)**	49.0 (18.5)^a^	54.1 (36.2)^a^
Trails B (s)	67.2 (29.9)^a^^,^^b^^,^^d^	**251.7 (73.1)**	**177.5 (86.6)**	168.5 (100.1)^a^	**168.6 (93.1)**^a^
*Language skills*					
WASI vocabulary (/80)	70.1 (4.9)	**51.9 (16.7)**	**22.5 (20.3)**^a^^,^^d^	**25.8 (20.1)**	**49.7 (19.2)**
BPVS (/150)	147.4 (1.0)	124.5 (29.2)	**109.9 (48.7)**	**64.1 (42.9)**^a^^,^^b^^,^^d^	122.4 (41.5)
GNT (/30)	25.1 (2.8)	**12.9 (8.0)**	**12.2 (7.2)**	**1.5 (4.5)**^a^^,^^b^^,^^d^	**14.6 (9.8)**
*Other skills*					
WASI Block Design (/71)	47.3 (12.4)	**12.6 (8.2)**^c^^,^^d^	**17.3 (16.0)**^c^^,^^d^	38.8 (17.3)	**32.4 (13.1)**
GDA (/24)	15.9 (4.9)	**4.5 (5.6)**^c^	**6.8 (7.5)**	12.1 (5.6)	**8.6 (6.1)**
VOSP Object Decision (/20)	18.2 (1.3)	15.7 (3.0)	**13.8 (5.0)**	15.4 (3.1)	15.6 (3.7)

Mean (standard deviation) values are shown unless otherwise indicated; scores that are statistically significantly different from healthy controls are shown in bold (maximum scores for neuropsychological tests are in parentheses).

AD, patient group with typical Alzheimer's disease; BPVS, British Picture Vocabulary Scale (Dunn et al., 1982); bvFTD, patient group with behavioural variant frontotemporal dementia; *C9orf72*, pathogenic mutation in open reading frame of chromosome 9; Controls, healthy control group; D-KEFS, Delis Kaplan Executive System (Fine & Delis, 2011); GDA, Graded Difficulty Arithmetic test (Jackson & Warrington, 1986); GNT, Graded Naming Test (McKenna & Warrington, 1980); *GRN*, pathogenic mutation in progranulin gene; *MAPT,* pathogenic mutation in microtubule-associated protein tau gene; MMSE, Mini-Mental State Examination score (Folstein et al., 1975); nfvPPA, patient group with nonfluent – agrammatic variant primary progressive aphasia; RMT, Recognition Memory Test (Warrington, 1984); svPPA, patient group with semantic variant primary progressive aphasia; Trails-making task based on maximum time achievable (2.5 min on task A, 5 min on task B) (Lezak et al., 2004); VOSP, Visual Object and Spatial Perception Battery – Object Decision test (Warrington & James, 1991); WASI, Wechsler Abbreviated Scale of Intelligence (Wechsler, 1997).

a Statistically significantly less than AD group.

b Statistically significantly less than nfvPPA group.

c Statistically significantly less than svPPA.

d Statistically significantly less than bvFTD (all P_bonf_<.05).

### 3.2 Identification of laughter subtypes

**Fig. 1:**
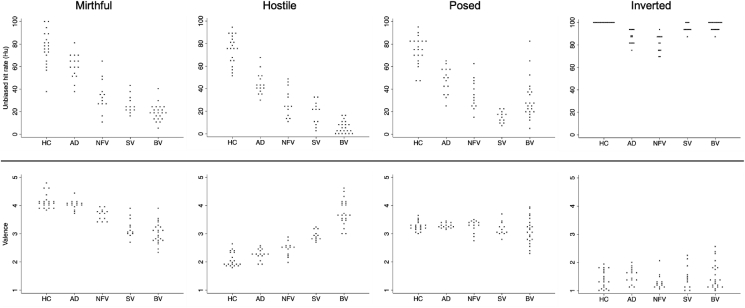
Individual data plots for identification accuracy and affective valuation for each laughter condition, across participant groups. The panels represent experimental laughter conditions (mirthful, hostile, posed) and the spectrally inverted laughter (inverted) control condition. Plotted on each panel are individuals' raw laughter identification accuracy scores (indexed as the unbiased hit rate) or affective valence ratings (on a 5-point Likert scale: 1, very unpleasant; 5, very pleasant) within each participant group. AD, patient group with typical Alzheimer's disease; BV, patient group with behavioural variant frontotemporal dementia; HC, healthy control group; NFV, patient group with nonfluent-agrammatic variant primary progressive aphasia; SV, patient group with semantic variant primary progressive aphasia.

**Fig. 2:**
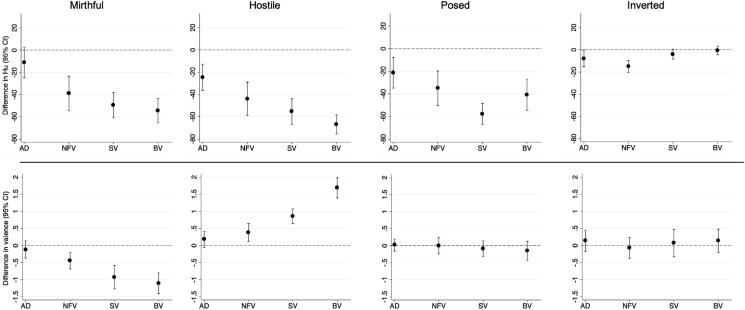
Identification accuracy and affective valuation of laughter conditions: patient groups versus healthy controls. The panels represent experimental laughter conditions (mirthful, hostile, posed) and the spectrally inverted laughter (inverted) control condition. Plotted on each panel are mean differences (with 95% confidence intervals, adjusted for multiple comparisons with Bonferroni correction of *p*-values) in unbiased hit rates [Hu] (top panels) or rated valence (bottom panels) between each patient group and the healthy control group. Numerical data are presented in Supplementary Tables S1 and S4. The horizontal dashed line on each panel indicates the zero level corresponding to no difference between patient group and heathy control group. AD, patient group with typical Alzheimer's disease; BV, patient group with behavioural variant frontotemporal dementia; HC, healthy control group; NFV, patient group with nonfluent-agrammatic variant primary progressive aphasia; SV, patient group with semantic variant primary progressive aphasia.

Laughter identification accuracy (hit rate) data for participant groups and experimental conditions are presented in Fig. 1 and Supplementary Table S1. There was strong evidence of main effects on unbiased laughter-identification hit rate of participant group [F(4) = 145.64; *p* < .001] and laughter condition [F(3) = 788.30; *p* < .001] and an interaction between them [F(12) = 77.10; *p* < .001]. Differences between each patient group and the healthy control group are presented in Fig. 2 and Supplementary Table S1. There was no statistically significant difference in overall laughter identification accuracy between male and female participants (*p* = .34) nor any significant correlation of age with laughter identification accuracy in this cohort (*r* = −.12, *p* = .22).

Mirthful laughter was more accurately identified by both the healthy control and AD groups than by all three FTD syndromic groups (all p_bonf_<.001); in all comparisons the magnitude of difference in unbiased hit rates was considerable, ranging from around 30 to 55%. There was no significant difference between the control and AD groups (p_bonf_ = .213). Hostile and posed laughter were more accurately identified by the healthy control group than by all patient groups (all p_bonf_<.001), with the magnitude of the deficit greatest for the bvFTD group for hostile laughter [−66.9% (CI −75.5, −58.3)] and for the svPPA group for posed laughter [−57.8% (CI −7.1, −48.5). Hostile and posed laughter were also more accurately identified by the AD group than by all three FTD groups (all p_bonf_<.05), although the magnitudes of difference were smaller (ranging from 14 to 42%). Comparing between FTD syndromic groups, the svPPA group was less accurate identifying posed laughter than both the nfvPPA and bvFTD groups whilst the bvFTD group was less accurate identifying hostile laughter than both the nfvPPA and svPPA groups – in all these comparisons, the magnitude of difference was around 20%. For identification of spectrally inverted laughter, the healthy control group performed at ceiling; the nfvPPA group was less accurate than all other groups (all p_bonf_<.04), with magnitudes of difference 8–15%), while the AD group was less accurate than both the healthy control and bvFTD groups (both p_bonf_<.03, with magnitudes of difference around 7%).

Within-group profiles comparing identification of different laughter conditions are detailed in Supplementary Table S2. In summary, all participant groups were more accurate identifying spectrally inverted laughter than all other laughter-subtypes (all p_bonf_<.001). In addition, the AD group was more accurate identifying mirthful laughter than hostile or posed laughter (both p_bonf_<.001); while the svPPA group was more accurate identifying mirthful laughter than posed laughter and the bvFTD group was less accurate identifying hostile laughter than all other laughter subtypes (all p_bonf_<.001).

**Fig. 3:**
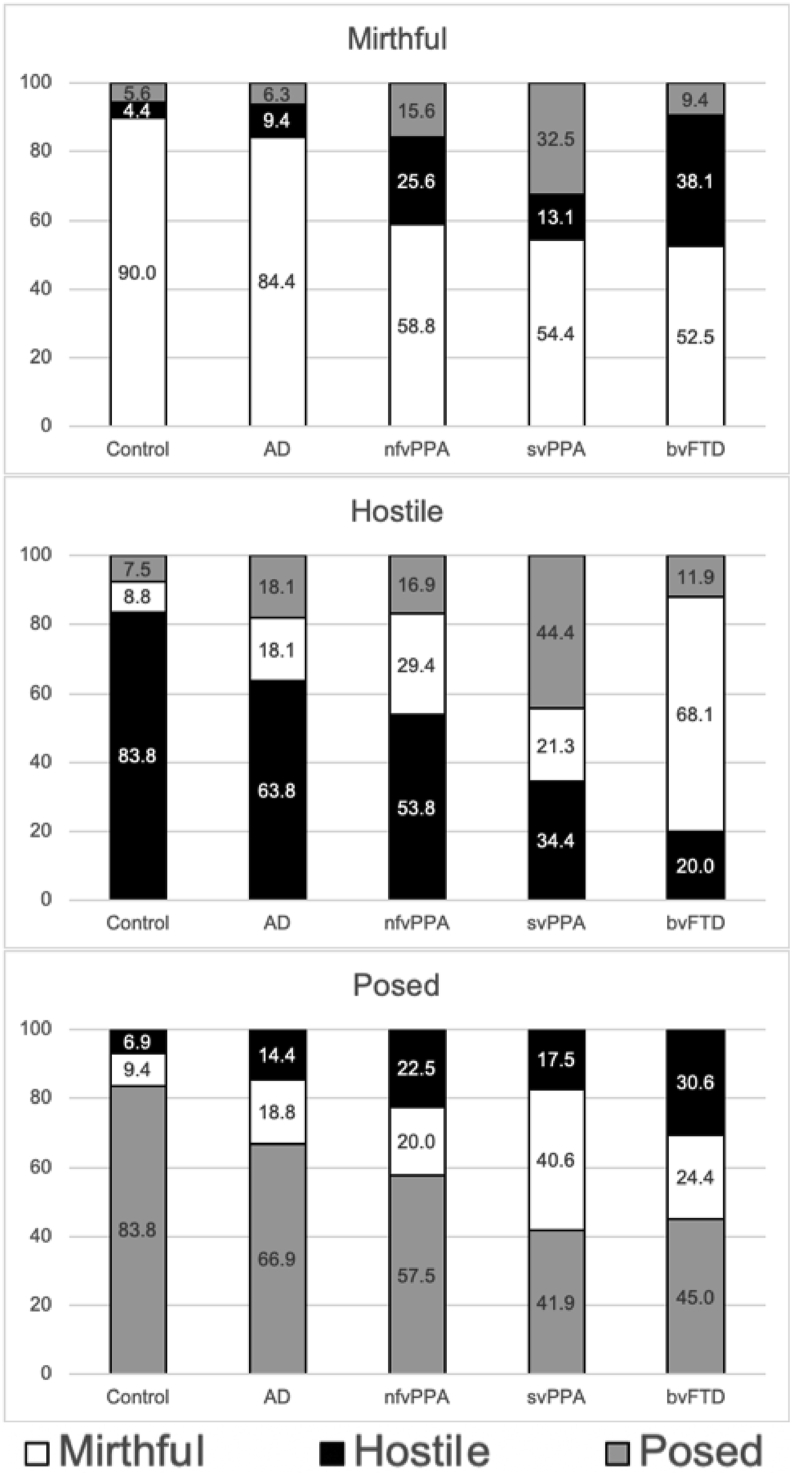
Cognitive labelling of each laughter condition, across participant groups. Participant group profiles of laughter labelling are shown in separate panels corresponding to each ‘target’ laughter condition. Percentages of each response given (averaged across all participants within each group) are indicated; response categories are coded as colours: white, ‘mirthful’; black, ‘hostile’; grey, ‘posed’. Raw data are presented in Supplementary Table S3. AD, patient group with typical Alzheimer's disease; bvFTD, patient group with behavioural variant frontotemporal dementia; Controls, healthy control group; nfvPPA, patient group with nonfluent-agrammatic variant primary progressive aphasia; SD, standard deviation; svPPA, patient group with semantic variant primary progressive aphasia.

Profiles of laughter identification and misidentification are presented in Fig. 3 and raw data on laughter confusion errors with odds ratios are presented in Supplementary Table S3. Most saliently, the bvFTD group confused mirthful with hostile laughter more often than did all other participant groups; while the svPPA group confused mirthful and hostile laughter with posed laughter more often than did all other participant groups.

### 3.3 Associations between laughter identification accuracy and other behavioural measures

Across the FTD cohort (20 bvFTD, 13 nfvPPA and 12 svPPA), controlling for age, audiometry score and WASI matrices score in a linear regression model, there was a statistically significant association between laughter identification score and both the mIRI (β = .47, *p* < .001) and RSMS (β = .32, *p* < .001). These relationships are illustrated in Supplementary Figure S1.

Across the patient population, there was also a statistically significant, yet weaker association between laughter identification and general semantic competence, as indexed by BPVS score (β = .17, *p* = .035).

### 3.4 Valence ratings of auditory stimuli

Perceived valence of auditory stimuli by participant group and sound condition are presented in Fig. 1 and Supplementary Table S4 whilst difference in valence scores between the patient groups and the healthy control group are illustrated in Fig. 2 and Supplementary Table S4. There was strong evidence of a main effect of experimental condition [F(4) = 618.77; *p* < .001] though not of participant group [F(4) = 2.41; *p* = .056]. There was however a significant interaction between the effect of participant group and experimental condition [F(16) = 45.73; *p* < .001].

Compared with the healthy control group, each of the FTD syndromic groups found mirthful laughter significantly less pleasant and hostile laughter significantly more pleasant (all p_bonf_≤.001); the greatest valence rating differences were between the healthy control and bvFTD groups: −1.11 (−1.41, −.80) for mirthful laughter and 1.68 (1.39, 1.98) for hostile laughter). There were no significant differences between the healthy control and AD groups (all p_bonf_>.3). The AD group found mirthful laughter significantly more pleasant than did each of the FTD syndromic groups and hostile laughter less pleasant than did the svPPA and bvFTD groups (all p_bonf_≤.001). Comparing between FTD syndromic groups, the nfvPPA group found mirthful laughter significantly more pleasant and hostile laughter less pleasant than did the svPPA and bvFTD groups, with the greatest valence rating difference between the nfvPPA and bvFTD groups for hostile laughter [1.31 (.97, 1.65)]. The bvFTD group also found hostile laughter significantly more pleasant than did the svPPA group [.83 (.54, 1.12)]. There were no statistically significant differences in valence ratings for posed or spectrally inverted laughter between the groups (all p_bonf_>.3).

**Fig. 4:**
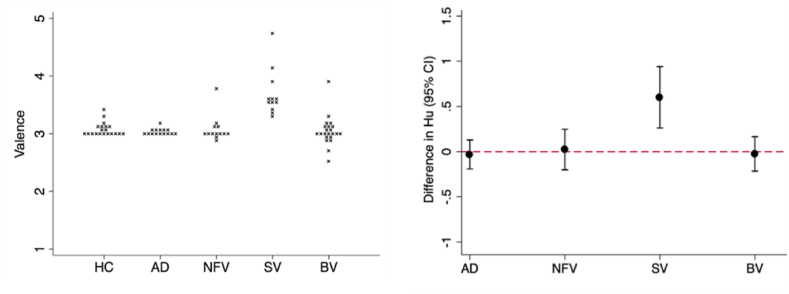
Affective valuation of spoken numbers by participant groups and in patients versus healthy controls. In the left panel, individual participants' average affective valence ratings of spoken numbers are plotted within each participant group. The right panel shows mean differences (with 95% confidence intervals, adjusted for multiple comparisons with Bonferroni correction of *p*-values) in rated valence between each patient group and the healthy control group; the horizontal dashed line indicates the zero level corresponding to no difference between patient group and heathy control group. AD, patient group with typical Alzheimer's disease; BV, patient group with behavioural variant frontotemporal dementia; HC, healthy control group; NFV, patient group with nonfluent-agrammatic variant primary progressive aphasia; SV, patient group with semantic variant primary progressive aphasia.

Of note, the svPPA group found the spoken number control condition significantly more pleasant than did all other groups (all p_bonf_≤.001), the magnitude of the valence rating difference being around .6 (see Fig. 4).

Within-group profiles are detailed in Supplementary Table S2. In summary, the healthy control, AD and nfvPPA groups found mirthful laughter significantly more pleasant than posed laughter and hostile laughter less pleasant than both mirthful and posed laughter. Conversely, the bvFTD group found hostile laughter significantly more pleasant than posed or mirthful laughter. All groups found spectrally inverted laughter the least pleasant sound. The svPPA group found spoken numbers significantly more pleasant than all other sounds apart from mirthful laughter.

### 3.5 Neuroanatomical associations of laughter identification

**Fig. 5:**
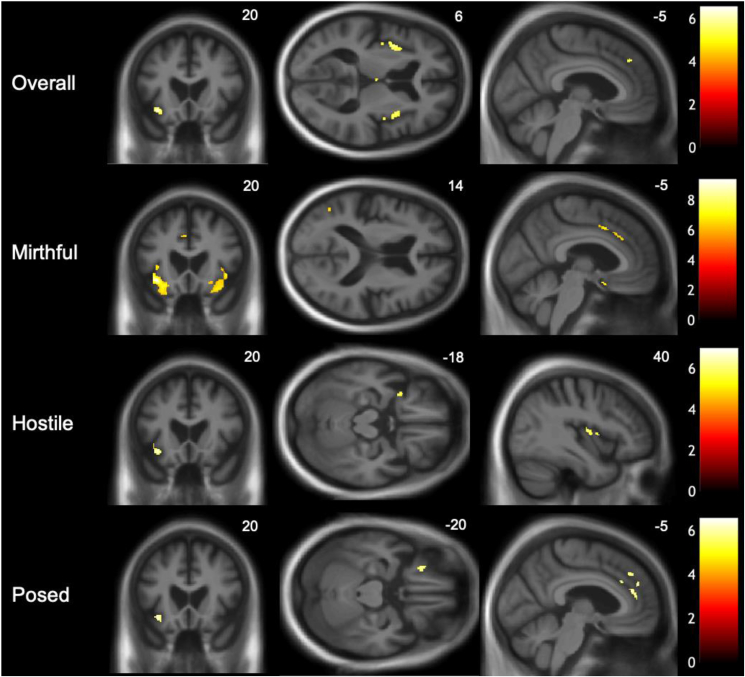
Neuroanatomical associations of laughter identification accuracy across the patient cohort. Statistical parametric maps (SPMs) of regional grey matter volume positively associated with overall laughter identification accuracy and accuracy of identification of particular laughter subtypes (derived from a voxel-based morphometric analysis) are shown for the combined patient cohort (see also Table 2). SPMs are overlaid on representative sections of the normalised study-specific T1-weighted group mean brain MR image, thresholded at *p* < .05_FWE_ corrected for multiple voxel-wise comparisons over the whole brain. The MNI coordinate (mm) of the plane of each section is indicated and the left cerebral hemisphere is shown on the left for coronal sections and at the top for axial sections; the colour bars code T values for each SPM.

Significant grey matter associations of overall laughter identification accuracy and unbiased hit rates for each laughter condition, across the entire patient cohort are summarised in Table 2; statistical parametric maps are presented in Fig. 5.

**Table 2:** Neuroanatomical associations of laughter identification in the combined patient cohort.

Contrast	Region	Side	Cluster (voxels)	Peak (mm)			T score	P_FWE_
				x	y	z		
Overall	Inferior frontal gyrus: pars orbitalis	L	119	−34	21	−12	7.29	<.001
	Anterior insula	L	183	−39	4	6	6.37	.002
		R	49	36	8	8	5.77	.010
	Posterior insula	L	18	−40	−6	8	5.61	.016
	Anteromedial prefrontal cortex	L	17	−4	39	40	5.78	.010
	Medio-dorsal thalamus	L	23	0	−16	3	5.69	.013
Mirthful	Inferior frontal gyrus: pars orbitalis	L	2350	−36	21	−10	9.41	<.001
	Anterior insula	R	998	42	21	−6	7.05	<.001
	Dorsal anterior cingulate cortex	L	33	−4	9	44	6.37	.002
	Posterior middle temporal gyrus	L	15	−50	−64	14	5.77	.01
Hostile	Anterior insula	L	267	−39	6	4	7.00	<.001
		R	11	40	3	6	5.46	.002
	Inferior frontal gyrus: pars orbitalis	L	84	−34	20	−12	6.80	<.001
	Posterior insula	R	27	40	−8	10	5.77	.01
	Orbitofrontal cortex	L	14	−33	12	−18	5.63	.015
Posed	Anteromedial prefrontal cortex	L	24	−3	45	28	6.57	<.001
			30	−4	39	40	5.68	.012
	Inferior frontal gyrus: pars orbitalis	L	50	−34	21	−12	6.35	.002
	Anterior cingulate cortex	L	64	−6	40	21	6.10	.003
	Orbitofrontal cortex	L	61	−27	28	−22	5.83	.008
	Anterior insula	L	47	−42	3	3	5.79	.009

Significant regional grey matter associations of overall laughter identification accuracy and unbiased hit rates (see text) for each laughter condition over the combined patient cohort, based on voxel-based morphometry. All clusters with extent larger than 10 voxels are shown. Coordinates of local maxima are in standard Montreal Neurological Institute space. *p* values were all significant (<.05) after family-wise error (FWE) correction for multiple voxel-wise comparisons over the whole brain.

Across the combined patient cohort, overall laughter identification accuracy was significantly positively associated (P_FWE_<.05 over the whole brain) with grey matter volume in the left pars orbitalis of inferior frontal gyrus, anteromedial prefrontal cortex, medio-dorsal thalamus and posterior insula and in bilateral anterior insula. Examining the neuroanatomical correlates of accurate identification of particular laughter subtypes in the combined patient cohort, unbiased hit rates for mirthful, hostile and posed laughter were all significantly positively associated (P_FWE_<.05 over the whole brain) with grey matter volume in the left pars orbitalis of inferior frontal gyrus and anterior insula. In addition, hit rates for mirthful laughter were significantly positively associated with grey matter in left dorsal anterior cingulate cortex and posterior middle temporal gyrus; hit rates for hostile laughter were significantly positively associated with grey matter in right posterior insula and left orbitofrontal cortex; while hit rates for posed laughter were significantly positively associated with grey matter in left anteromedial prefrontal, anterior cingulate and orbitofrontal cortices.

## 4 Discussion

**Fig. 6:**
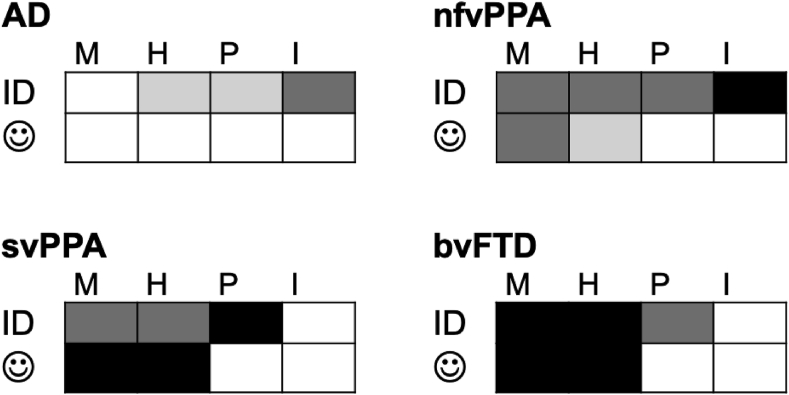
Syndromic profiles of cognitive and affective responses to laughter. The figure summarises the cognitive (laughter identification, **ID**) and affective (valence rating, face icon) responses to laughter subtypes (**M**, mirthful; **H**, hostile; **P**, posed; **I**, spectrally inverted) in the four dementia syndromes studied here. Shaded cells indicate a significantly abnormal alteration of laughter processing, coded as follows: white, no impairment; light grey, relative to healthy controls; dark grey, relative to the other disease group (FTD *vs* AD); black, relative to other syndromes within the FTD spectrum (see text and Supplementary Tables S1 and S4 for details). AD, patient group with typical Alzheimer's disease; bvFTD, patient group with behavioural variant frontotemporal dementia; nfvPPA, patient group with nonfluent-agrammatic variant primary progressive aphasia; svPPA, patient group with semantic variant primary progressive aphasia.

Relative to healthy older individuals, patients with major syndromes of FTD and AD exhibit richly differentiated profiles of impaired cognitive and affective processing of laughter. These profiles are summarised graphically in Fig. 6. While all dementia syndromes demonstrated impaired identification of laughter subtypes, this was more severe overall in FTD syndromes (particularly svPPA and bvFTD) than in AD. A qualitatively similar differentiation was found for the affective evaluation of laughter: this was normal in AD but severely affected in bvFTD and svPPA. Dementia syndromes were further stratified based on the processing of particular laughter subtypes. Impaired processing of mirthful and hostile laughter was a hallmark of FTD syndromes compared with both healthy controls and patients with AD. The bvFTD group in particular frequently confused mirthful and hostile laughter and demonstrated an abnormal liking for unpleasant (hostile) laughter. Impaired processing of synthetic (spectrally inverted) laughter-like signals was a hallmark of nfvPPA relative to other participant groups; while enhanced liking for a non-affective vocal signal (spoken numbers) over laughter was a striking feature of svPPA. Impaired processing of laughter in the patient cohort was underpinned by regional grey matter atrophy in distributed cerebral networks encompassing inferior and orbitofrontal, cingulate, insular, posterior temporal and anteromedial prefrontal cortices.

The panoply of ‘laughter phenotypes’ in different dementia syndromes is consistent with the diverse behavioural ends that laughter serves in everyday communication and with other signal processing deficits previously described in these canonical dementias. The severely impaired comprehension of laughter as an emotional and social signal in the bvFTD and svPPA groups here accords with the well-documented difficulty these patients have with understanding and responding appropriately to many kinds of social and emotional signals, including elementary emotional expressions, sarcasm and mental state attribution (Bertoux et al., 2016; Caminiti et al., 2015; Downey et al., 2013; Hsieh et al., 2013; Hutchings et al., 2017; Keane et al., 2002; Marshall et al., 2019; Rohrer et al., 2012; Rosen et al., 2004; Snowden et al., 2008). Indeed, the capacity to understand laughter as a socio-emotional signal may predict daily-life socio-emotional reactivity, as evidenced by the strong positive correlation of laughter identification accuracy with mIRI and RSMS scores in the svPPA and bvFTD groups here. Whereas impaired processing of ‘negatively’ valenced emotions has been emphasised in previous neuropsychological studies of FTD (Hsieh et al., 2013; Keane et al., 2002; Kumfor & Piguet, 2012; Lavenu et al., 1999; Lough et al., 2006), this might reflect a bias inherent in standard instruments such as the Ekman faces, which comprise four negative elementary emotions but only a single prototypical positive emotion (happiness). This skewed over-representation of negatively-valenced relative to positively-valenced emotions in most studies of emotion recognition may have led to valence-incongruent errors being under-recognised in dementia syndromes (Bertoux et al., 2020). Our findings suggest that the cognitive differentiation of perceptually related emotional signals (rather than their valence per se) challenges dysfunctional mechanisms of emotion decoding in FTD.

It is noteworthy that the identification of posed laughter here was abnormal across the dementia syndromes, and not restricted to those groups with more severe difficulty judging authenticity of others’ emotions in daily life (namely svPPA and bvFTD). However, judgements about laughter authenticity are likely to depend sensitively on accurate perceptual encoding as well as social cognitive decoding, and the mechanism of impairment is likely to have varied between the syndromes. The severe deficit in the svPPA group here is consistent with other evidence for impaired mentalising, affective semantic and social conceptual decoding in this syndrome (Bejanin et al., 2017; Clark et al., 2017; Irish et al., 2014; Zahn et al., 2017), amplified in situations that call for resolution of ambiguity or conflict. Indeed, both recognition of emotional facial expressions and knowledge of emotional concepts are impaired in svPPA and furthermore, cross-valence errors in this disorder have been shown to correlate with emotion conceptual knowledge, suggesting that semantic knowledge may guide not only the recognition of emotions but also valence assignment (Bertoux et al., 2020) By contrast, in nfvPPA, our findings suggest that the perception of complex spectrotemporal signals is fundamentally abnormal, building on emerging evidence for a generic disorder of acoustic analysis in this syndrome (Goll et al., 2010; Hardy et al., 2017a, 2017b, 2019; Rohrer et al., 2012). A fundamental impairment of vocal perceptual analysis would potentially also account for the frequent confusion of mirthful and hostile laughter by the nfvPPA group, as these laughter conditions here were acoustically rather similar (see Supplementary Table S5). Moreover, natural laughter is usually accompanied by various other contextual cues that patients with nfvPPA may be able to exploit in their daily lives.

While impaired cognitive labelling of laughter subtypes was accompanied by alterations in affective evaluation across the patient cohort, these two dimensions of laughter processing did not correlate simply within particular dementia syndromes; rather, there was evidence for substantial dissociation. Patients with AD showed normal affective evaluation of all laughter subtypes and even patients representing FTD syndromes showed normal affective evaluation of posed and inverted laughter, despite deficient cognitive labelling. Altered hedonic behaviours in response to environmental sounds and music in daily life are frequently reported in FTD syndromes (in particular bvFTD and svPPA) as well as AD (Fletcher et al., 2015a) and abnormal affective evaluation of music has been described in bvFTD and svPPA (Clark et al., 2018), while another study found that explicit affective valuation of environmental sounds may be normal in these syndromes (Fletcher et al., 2015b). The strikingly abnormal affective preference for hostile over mirthful laughter in the bvFTD group here is in keeping with other evidence that these patients may find humour in frankly inappropriate or unpleasant situations (Clark et al., 2015, 2016), and may have contributed to the frequent confusion between hostile and mirthful laughter in this group.

The svPPA group here uniquely rated spoken numbers as more pleasant than most laughter subtypes – this ‘numerophilia’ may reflect a shift in hedonic drive toward inanimate stimuli akin to the behavioural repertoire of sometimes obsessive, impersonal preoccupations and interests exhibited by patients with syndromes of focal temporal lobe atrophy, which often includes mathematical puzzles (Chan et al., 2009; Fletcher et al., 2015a; Green & Patterson, 2009; Papagno et al., 2013; Sivasathiaseelan et al., 2019). Taken together, this evidence paints a complex picture of dissociable linkages between different dimensions of complex auditory signal analysis in canonical dementias.

The neuroanatomical substrates for overall accuracy identifying laughter in our patient cohort centred on a common, distributed fronto-cingulo-insular network previously implicated in processing and resolving ambiguity in emotional sounds including human socio-emotional signals and more particularly, laughter (Frühholz et al., 2014, 2016) Fronto-cingulo-insular circuitry appraises the salience of sensory stimuli and prepares contextually appropriate behavioural responses (Levy & Wagner, 2011). The anterior insula hosts an interface between sensory, affective and cognitive brain systems that process emotional sounds (Bamiou et al., 2003; Fruhholz & Grandjean, 2012; Kumar et al., 2012; Mirz et al., 2000; Sander & Scheich, 2005; Trost et al., 2012). Within the inferior frontal cortex, pars orbitalis acts as a hub zone for the cognitive and affective decoding of auditory signals (Belyk et al., 2017), particularly where these constitute patterns bound by ‘rules’ and expectancies. Besides its well-known role in linguistic grammar processing, this region is involved in processing musical syntax (Maess et al., 2001) and affective evaluation of harmonic progressions in melodies (Clark et al., 2018). Anteromedial prefrontal and anterior cingulate cortices behave as an integrated functional ‘hub’ in appraising the social value of heard laughter and disambiguating its social intent (Ethofer et al., 2020; Lavan et al., 2017; McGettigan et al., 2015) and programming adaptive output behaviours, including own laughter and the subjective experience of mirth (Beckmann et al., 2009; Touroutoglou & Dickerson, 2019; Touroutoglou et al., 2020; Vogt, 2005; Yu et al., 2011). Further correlates of overall laugher identification accuracy were identified here in closely structurally and functionally interconnected regions that are likely to be obligatorily engaged in appraising and responding to laughter: posterior insula, essential for integrating interoceptive information (Wattendorf et al., 2019) and key acoustic cues that convey emotional content (Patel et al., 2011; Sauter et al., 2010a; Schirmer & Kotz, 2006; Zhang et al., 2019) during behavioural preparation; and mediodorsal thalamus, implicated in cognitive set shifting to meet changing behavioural contingencies (Vertes et al., 2015).

In line with its core role in the analysis of salient auditory signals, anterior insular and inferior frontal circuitry was correlated here with accuracy identifying all laughter subtypes when these were examined separately. Additionally, more specific cortical associations were delineated for the identification of particular laughter subtypes. Accurate identification of mirthful laughter was additionally linked to posterior middle temporal gyrus, a region previously implicated in the processing of sensory ‘templates’ for humour (Clark et al., 2015). Identification of hostile laughter was additionally linked to posterior insula (as anticipated for a sensory signal with powerful homeostatic resonance) and orbitofrontal cortex, integral to the resolution of conflict and ambiguity in social signals based on hedonic and behavioural cues (Beyer et al., 2015; Clark et al., 2017, 2018; Kringelbach, 2005; Kringelbach & Rolls, 2004; Strenziok et al., 2011). Identification of posed laughter – a paradigmatic ‘socially ambiguous’ vocalisation – was additionally linked both to orbitofrontal cortex and an anteromedial prefrontal cortical region previously proposed to engage in obligatory mentalising during the evaluation of laughter authenticity and intent (McGettigan et al., 2015). These condition-specific associations illustrate the potency of laughter as a probe of social brain mechanisms. The human social brain connectome principally comprises four hierarchically interlocking neural networks (Alcalá-López et al., 2017): a ‘sensory’ network mediating analysis of auditory features and patterns, here represented by the thalamic, posterior insular and inferior frontal correlates of general laughter identification; a ‘limbic’ (mesial temporal – ventromedial prefrontal) network mediating affective disambiguation of stimuli, here represented by the orbitofrontal correlates of hostile and posed laughter identification; an ‘intermediate’ (cingulo-insular) network integrating salient environmental and bodily states, here represented across laughter subtypes; and a ‘higher associative’ (temporo-parietal – dorsomedial prefrontal) network engaged in decoding mental states, here represented by the identification of mirthful and posed laughter.

This study has several limitations that should direct future work. Larger patient cohorts with histopathological and molecular correlation and autonomic, electrophysiological and dynamic neuroimaging techniques that can capture functional changes in neural networks will ultimately be required to define fully the pathophysiological phenotypes delineated here (Perry et al., 2017). Work of this kind stands sorely in need of a validated measure of general disease severity that could be incorporated as a nuisance covariate in group comparisons across the AD and FTD spectrum and which is not heavily confounded by linguistic impairment; here, we adopted the WASI Matrices score as a non-verbal, multi-componential measure, however a composite of several test scores may be a more appropriate target.

The interface of laughter processing with the processing of other vocal signals, and between the autonomic, affective, semantic and social conceptual dimensions of this highly complex socioemotional signal should be explored comprehensively, This is likely to require indices of emotion-specific conceptual knowledge (Bertoux et al., 2020). The extent to which laughter identification deficits correlate with deficits in other, standardised measures of social and emotional cognition also merits further investigation. Functional and connectivity-based neuroimaging techniques such as fMRI and MEG are likely to be particularly important for delineating the neural correlates of affective and reward processing that are intrinsically dynamic, nonlinear, anatomically distributed and challenging to quantify behaviourally.

In this first study of its kind in dementia, we have been deliberately reductionist in our deconstruction of the putative components of laughter processing. However, laughter signals in everyday life are deployed on an affective and semantic continuum that is exquisitely sensitive to social and homeostatic context. Appropriate integration of laughter characteristics with contextual factors is likely to be essential to successful socio-emotional functioning and these integrative processes may well be targeted in neurodegenerative disease. Future studies of laughter signalling in clinical populations will ultimately need to grapple with this issue and address the potential effects of listener as well as sender characteristics and their interaction (Szameitat et al., 2011).

As an intrinsically ambiguous stimulus (Anikin & Persson, 2017), laughter taxes neural perceptual and socio-emotional processing mechanisms and therefore might constitute a ‘stress test’ or ‘cognitive marker’ for early detection and tracking of reduced processing fidelity in neurodegenerative proteinopathies: however, this will only be confirmed with longitudinal studies, ideally including presymptomatic mutation carriers. From a more practical standpoint, multi-centre, international studies addressing social cognition in dementia populations could exploit the non-linguistic status of laughter. Whilst cultural and socio-economic factors are very likely to influence how laughter is used and interpreted (de Souza et al., 2018), it is nevertheless a universal human socio-emotional signal of high behavioural salience (Bryant et al., 2016, 2018; Sauter et al., 2010b). In everyday life, however, laughter does not occur in the disembodied form presented here but embedded in a social context: neuropsychological deficits of laughter processing will need to be assessed in relation to such contextual factors as well as behavioural symptoms, in order to fully evaluate laughter as an index of social-emotional dysfunction in dementia. Our findings (in particular, the indexing of daily life socio-emotional competence by laughter identification accuracy) present a strong prima facie case for further studies of laughter processing incorporating additional measures of social cognition and daily life functioning in people living with dementia.

## Author contributions

**Harri Sivasathiaseelan:** Conceptualisation, Methodology, Investigation, Formal analysis, Writing – Original draft preparation, Writing – Reviewing and Editing; **Charles R Marshall:** Methodology, Investigation, Writing – Reviewing and Editing; **Elia Benhamou:** Methodology, Investigation, Writing – Reviewing and Editing; **Janneke EP van Leeuwen:** Methodology, Writing – Reviewing and Editing; **Rebecca L Bond:** Investigation, Writing – Reviewing and Editing; **Lucy L Russell:** Investigation, Writing – Reviewing and Editing; **Caroline Greaves:** Investigation, Writing – Reviewing and Editing; **Katrina Moore:** Investigation, Writing – Reviewing and Editing; **Chris JD Hardy:** Methodology, Investigation, Writing – Reviewing and Editing; **Chris Frost:** Formal analysis, Writing – Reviewing and Editing; **Jonathan D Rohrer:** Methodology, Writing – Reviewing and Editing, Supervision; **Sophie K Scott:** Conceptualisation, Methodology, Writing – Reviewing and Editing, Supervision; **Jason D Warren:** Conceptualisation, Methodology, Writing – Reviewing and Editing, Supervision.

## Funding

The Dementia Research Centre is supported by Alzheimer's Research UK, the Brain Research Trust and the Wolfson Foundation. This work was funded by the Alzheimer's Society, Leonard Wolfson Experimental Neurology Centre, Medical Research Council UK, and the NIHR UCLH Biomedical Research Centre. HS and CRM were supported by Clinical Research Fellowships from the Leonard Wolfson Experimental Neurology Centre. EB is supported by a Brain Research UK Ph.D. Studentship. JEPL was funded by an EPSSRC Ph.D. Studentship. RLB was funded by an MRC Ph.D. Studentship. KMM was supported by a grant from the Alzheimer's Society. CJDH is a Pauline Ashley Action on Hearing Loss and Dunhill Medical Trust Postdoctoral Fellow. CF's academic collaboration with the Dementia Research Centre, UCL is supported by a grant from Alzheimer's Research UK. JDR is supported by an MRC Clinician Scientist Fellowship and has received funding from the NIHR Rare Disease Translational Research Collaboration.

## Declaration of competing interest

The authors report no competing interests.

No part of the study procedures was pre-registered prior to the research being conducted.

No part of the study analyses was pre-registered prior to the research being conducted.
