# Supplementary material for: Laughter as a paradigm of socio-emotional signal processing in dementia
Source: Cortex. 2021 Sep;142:186–203. doi: 10.1016/j.cortex.2021.05.020 (PMC8438290; doi:10.1016/j.cortex.2021.05.020)
Supplement: Multimedia component 1 [file mmc1.docx]

## Supplementary Material: Laughter as a paradigm of socio-emotional signal processing in dementia, by H Sivasathiaseelan et al.

**Laughter stimulus selection**

Stimuli representing the three major natural laughter categories of interest (mirthful, hostile and posed) were obtained from two main sources. Examples of mirthful and posed laughter were generated by the Speech Communications Lab at University College London (McGettigan et al. 2015). Further examples of mirthful and posed laughter and examples of hostile laughter were obtained from online videos ([www.youtube.com](http://www.youtube.com)). Laughter was deemed mirthful when it occurred spontaneously in response to an unequivocally amusing situation in the context of a social interaction, often between friends. Hostile laughter was derived from a variety of situations involving the spectating of another’s misfortune, such as onlookers laughing at another’s failed attempt at a stunt and individuals taunting each other whilst playing sports. Examples of posed laughter were gathered from videos in which the individual clearly produced the laughter volitionally. e.g. an actor demonstrating how they can portray laughter with no stimulus or friends testing whether they can identify each other’s faked laughter.

The audio track from each clip was extracted and edited to ensure that the final laughter stimulus did not contain extraneous vocal or environmental noises. All samples of laughter were derived from younger adult males, to avoid any potentially confounding effect from age or gender on perceived laughter intent. Laughter recordings were converted in MatlabR14® to digital WAV files with sampling rate 44.1 kHz, 32-bit resolution, uniform root-mean-square intensity and duration around two seconds (range 1.9 - 2.2 seconds).

In an initial pilot experiment, 75 examples of laughter stimuli (25 for each laughter condition) were presented to 12 healthy, young adults (median age 26, range 22-33, six female) who were asked to identify the kind of laughter represented by each stimulus. Stimuli that were misidentified by more than three (25%) of the pilot group were excluded. Participants in the pilot experiment rated the authenticity of each laughter stimulus on a 5-point Likert scale (1=definitely posed, 5=definitely genuine), in order to allow us to select the 16 best examples of each laughter condition (genuine emotional – mirthful or hostile – and posed laughter) for inclusion in the final stimulus set. The laughter stimuli presented in the main experiment were thus highly identifiable and representative examples of different types of laughter that could be heard in daily life. Posed laughter is, of course, by definition ‘artificial’: for the purpose of this experiment, the key issue was to distinguish this stimulus condition from the conditions based on spontaneous (emotionally genuine) laughter.

In this pilot experiment, participants also listened to 16 examples of spectrally inverted laughter and spoken numbers and were asked to rate their valence on a 5-point Likert scale (1=very unpleasant, 5=very pleasant). The mean valence rating for inverted laughter was 1.58 (95% confidence interval 1.39-1.77) and for spoken numbers was 3.02 (95% confidence interval 2.93-3.11), confirming the aversive nature of inverted laughter and the affectively ‘neutral’ quality of spoken numbers.

An example of each stimulus condition is included in Supplementary Material online. Acoustic details of the stimulus conditions are summarised in **Supplementary Table S5**.

**Audiometry protocol: assessment of peripheral hearing function**

Using an Otovation Roto® audiometer (https:/www.auditdata.com/) with a single TDH-39P 10-ohm Telephonics® earphone (www.telephonics.com) in a quiet room, steady tones of 500, 1000, 2000, 4000 and 6000Hz were presented separately to each of the participant’s ears separately, over ascending intensity levels commencing at 20dB HL (decibel hearing level). At each frequency, the participant indicated (verbally or by gesture) when they first heard a noise. If the participant was unable to hear the tone, the level was increased in 5dB increments (maximum 70dB HL). This procedure was repeated three times to generate a mean threshold for that frequency. For each participant a composite hearing threshold score was created by calculating the mean threshold across all frequencies in the best ear.

**Supplementary Table S1.** Unbiased hit rates for identification of laughter subtypes and comparisons between participant groups

| **Condition** | **Group** | **Hu**  Mean (SD) | **Controls** | | **AD** | | **nfvPPA** | | **svPPA** | |
| --- | --- | --- | --- | --- | --- | --- | --- | --- | --- | --- |
|  |  |  | Difference (CI) | P | Difference (CI) | P | Difference (CI) | P | Difference (CI) | P |
| **Mirthful** | Controls | 76.1 (15.1) |  |  |  |  |  |  |  |  |
|  | AD | 59.7 (11.4) | -11.2 (-24.9, 2.6) | 0.213 |  |  |  |  |  |  |
|  | nfvPPA | 33.8 (14.6) | **-38.8 (-54.3, -23.3)** | **<0.001** | **-27.7 (-41.2, -14.1)** | **<0.001** |  |  |  |  |
|  | svPPA | 26.1 (7.9) | **-49.4 (-60.7, -38.1)** | **<0.001** | **-38.3 (-49.3, -27.3)** | **<0.001** | -10.6 (-23.6, 2.4) | 0.210 |  |  |
|  | bvFTD | 19.4 (7.5) | **-54.5 (-65.5, -43.6)** | **<0.001** | **-43.4 (-53.1, -33.6)** | **<0.001** | **-15.7 (-27.8, -3.6)** | **0.003** | -5.1 (-13.2, 3.1) | 0.750 |
| **Hostile** | Controls | 74.6 (12.7) |  |  |  |  |  |  |  |  |
|  | AD | 44.8 (9.5) | **-24.6 (-36.4, -12.9)** | **<0.001** |  |  |  |  |  |  |
|  | nfvPPA | 27.2 (12.3) | **-44.0 (-58.8, -29.1)** | **<0.001** | **-19.3 (-31.8, -6.9)** | **<0.001** |  |  |  |  |
|  | svPPA | 18.7 (10.5) | **-55.2 (-67.0, -43.6)** | **<0.001** | **-30.7 (-43.0, -18.4)** | **<0.001** | -11.3 (-26.5, 3.8) | 0.342 |  |  |
|  | bvFTD | 5.5 (4.9) | **-66.9 (-75.5, -58.3)** | **<0.001** | **-42.3 (-50.4, -34.2)** | **<0.001** | **-23.0 (-34.7, -11.2)** | **<0.001** | **-11.6 (-21.5, -1.8)** | **0.010** |
| **Posed** | Controls | 73.9 (12.5) |  |  |  |  |  |  |  |  |
|  | AD | 47.4 (11.9) | **-21.2 (-34.8, -7.6)** | **<0.001** |  |  |  |  |  |  |
|  | nfvPPA | 35.4 (13.1) | **-35.0 (-50.3, -20.0)** | **<0.001** | **-13.8 (-27.4, -0.3)** | **0.043** |  |  |  |  |
|  | svPPA | 15.5 (5.0) | **-57.8 (-67.1, -48,5)** | **<0.001** | **-36.6 (-47.8, -25.3)** | **<0.001** | **-22.7 (-35.7, -9.8)** | **<0.001** |  |  |
|  | bvFTD | 30.9 (17.7) | **-40.8 (-54.6, -27.1)** | **<0.001** | **-19.6 (-33.1, -6.1)** | **0.001** | -5.8 (-20.7, 9.2) | 1.00 | **17.0 (5.3, 28.6)** | **0.001** |
| **Inverted** | Controls | 100 (0) |  |  |  |  |  |  |  |  |
|  | AD | 86.9 (6.1) | **-7.9 (-15.2, -0.5)** | **0.028** |  |  |  |  |  |  |
|  | nfvPPA | 81.5 (7.9) | **-15.1 (-20.4, -9.7)** | **<0.001** | **-7.2 (-14.0, -0.4)** | **0.031** |  |  |  |  |
|  | svPPA | 95.3 (3.9) | -4.0 (-8.5, 0.5) | 0.122 | 3.9 (-4.0, 11.7) | 1.00 | **11.1 (5.0, 17.1)** | **<0.001** |  |  |
|  | bvFTD | 97.2 (3.7) | -0.6 (-4.4, 3.1) | 1.00 | **7.3 (0.8, 13.8)** | **0.018** | **14.5 (9.2, 19.7)** | **<0.001** | 3,4 (-1.7, 8.5) | 0.591 |

Mean (standard deviation) and adjusted differences between groups (with 95% confidence intervals) are shown for unbiased hit rate (Hu) of each laughter condition. P values and CI are Bonferroni corrected for multiple pairwise comparisons. Significant (p_bonf_<0.05) are shown in bold. AD, patient group with typical Alzheimer’s disease; bvFTD, patient group with behavioural variant frontotemporal dementia; CI, 95% confidence intervals; Controls, healthy control group; Inverted, spectrally inverted laughter control condition; nfvPPA, patient group with nonfluent-agrammatic variant primary progressive aphasia; SD, standard deviation; svPPA, patient group with semantic variant primary progressive aphasia.

**Supplementary Table S2**. Within-group comparisons of unbiased hit rates and valence ratings between experimental conditions

| **Comparison** | **Controls** | | **AD** | | **nfvPPA** | | **svPPA** | | **bvFTD** | |
| --- | --- | --- | --- | --- | --- | --- | --- | --- | --- | --- |
|  | *Difference (CI)* | *P* | *Difference (CI)* | *P* | *Difference (CI)* | *P* | *Difference (CI)* | *P* | *Difference (CI)* | *P* |
| **Laughter identification** | | | | | | | | | | |
| Mirthful vs Hostile | 1.5 (-5.8, 8.7) | 1.00 | **14.9 (5.5, 24.3)** | **<0.001** | 6.6 (-4.4, 17.7) | 0.652 | 7.3 (-0.4, 15.1) | 0.076 | **13.9 (9.0, 18.8)** | **<0.001** |
| Mirthful vs Posed | 2.3 (-5.2, 9.7) | 1.00 | **12.3 (5.2, 19.4)** | **<0.001** | -1.5 (-13.2, 10.1) | 1.00 | **10.6 (5.8, 15.4)** | **<0.001** | -11.4 (-23.1, 0.2) | 0.058 |
| Mirthful vs Inverted | **-23.9 (-33.1, -14.6)** | **<0.001** | **-27.1 (-35.7, -18.5)** | **<0.001** | **-47.6 (-58.5, -36.8)** | **<0.001** | **-69.3 (-75.2, -63.4)** | **<0.001** | **-77.8 (-83.4, -72.1)** | **<0.001** |
| Hostile vs Posed | 0.8 (-8.6, 10.2) | 1.00 | -2.6 (-11.2, 6.0) | 1.00 | **-8.1 (-14.1, -2.2)** | **0.002** | 3.3 (-3.8, 10.3) | 1.00 | **-25.3 (-35.6, -15.0)** | **<0.001** |
| Hostile vs Inverted | **-25.4 (-33.2, -17.5)** | **<0.001** | **-42.1 (-50.5, -33.7)** | **<0.001** | **-54.2 (-64.4, -44.1)** | **<0.001** | **-76.6 (-85.7, -67.6)** | **<0.001** | **-91.7 (-95.2, -88.1)** | **<0.001** |
| Posed vs Inverted | **-26.1 (-33.8, -18.5)** | **<0.001** | **-39.5 (-47.9, -31.0)** | **<0.001** | **-46.1 (-56.6, -35.6)** | **<0.001** | **-79.9 (-84.1, -75.6)** | **<0.001** | **-66.3 (-76.2, -56.5)** | **<0.001** |
| **Laughter valence rating** | | | | | | | | | | |
| Mirthful vs Hostile | **2.05 (1.81, 2.28)** | **<0.001** | **1.75 (1.59, 1.91)** | **<0.001** | **1.23 (0.99, 1.48)** | **<0.001** | **0.27 (0.04, 0.50)** | **0.009** | **-0.74 (-1.06, -0.43)** | **<0.001** |
| Mirthful vs Posed | **0.87 (0.70, 1.03)** | **<0.001** | **0.73 (0.57, 0.89)** | **<0.001** | **0.42 (0.16, 0.68)** | **<0.001** | 0.03 (-0.18, 0.24) | 1.00 | -0.09 (-0.41, 0.23) | 1.00 |
| Mirthful vs Inverted | **2.73 (2.46, 3.00)** | **<0.001** | **2.47 (2.22, 2.72)** | **<0.001** | **2.36 (2.15, 2.57)** | **<0.001** | **1.73 (1.42, 2.04)** | **<0.001** | **1.48 (1.11, 1.86)** | **<0.001** |
| Mirthful vs Numbers | **1.06 (0.88, 1.24)** | **<0.001** | **0.97 (0.82, 1.13)** | **<0.001** | **0.59 (0.31, 0.87)** | **<0.001** | -0.47 (-0.94, 0.01) | 0.059 | -0.02 (-0.32, 0.28) | 1.00 |
| Hostile vs Posed | **-1.18 (-1.34, -1.02)** | **<0.001** | **-1.02 (-1.20, -0.84)** | **<0.001** | **-0.81 (-1.01, -0.61)** | **<0.001** | **-0.24 (-0.42, -0.06)** | **0.003** | **0.65 (0.34, 0.96)** | **<0.001** |
| Hostile vs Inverted | **0.68 (0.41, 0.95)** | **<0.001** | **0.72 (0.53, 0.91)** | **<0.001** | **1.13 (0.96, 1.30)** | **<0.001** | **1.46 (1.20, 1.72)** | **<0.001** | **2.22 (1.88, 2.57)** | **<0.001** |
| Hostile vs Numbers | **-0.99 (-1.19, -0.79)** | **<0.001** | **-0.78 (-0.93, -0.63)** | **<0.001** | **-0.63 (-0.91, -0.37)** | **<0.001** | **-0.74 (-1.12, -0.35)** | **<0.001** | **0.72 (0.41, 1.03)** | **<0.001** |
| Posed vs Inverted | **1.86 (1.62, 2.10)** | **<0.001** | **1.74 (1.51, 1.98)** | **<0.001** | **1.93 (1.69, 2.19)** | **<0.001** | **1.70 (1.38, 2.02)** | **<0.001** | **1.57 (1.25, 1.90)** | **<0.001** |
| Posed vs Numbers | **0.19 (0.06, 0.33)** | **0.001** | **0.24 (0.17, 0.32)** | **<0.001** | 0.17 (0.01, 0.43) | 0.608 | **-0.50 (-0.88. -0.12)** | **0.003** | 0.07 (-0.225, 0.39) | 1.00 |
| Inverted vs Numbers | **-1.67 (-1.91, -1.43)** | **<0.001** | **-1.50 (-1.70, -1.30)** | **<0.001** | **-1.77 (-2.03, -1.50)** | **<0.001** | **-2.20 (-2.76, -1.64)** | **<0.001** | **-1.50 (-1.83, -1.17)** | **<0.001** |

Differences (with 95% confidence intervals) are shown for identification accuracy (indexed as unbiased hit rate) and valence of each sound condition within each group. P values and CI are Bonferroni corrected for multiple pairwise comparisons. Statistically significant differences (p_bonf_<0.05) are shown in bold. AD, patient group with typical Alzheimer’s disease; bvFTD, patient group with behavioural variant frontotemporal dementia; CI, 95% confidence intervals; Controls, healthy control group; Inverted, spectrally inverted laughter control condition; nfvPPA, patient group with nonfluent-agrammatic variant primary progressive aphasia; Numbers, spoken numbers control condition; svPPA, patient group with semantic variant primary progressive aphasia.

**Supplementary Table S3.** Summary of error analysis: laughter condition confusions and comparisons between participant groups

| **Group** | **Error number (SD)** | **Control** | | | **AD** | | | **nfvPPA** | | | **svPPA** | | |
| --- | --- | --- | --- | --- | --- | --- | --- | --- | --- | --- | --- | --- | --- |
|  |  | OR | CI | P | OR | CI | P | OR | CI | P | OR | CI | P |
| **Mirthful misidentified as hostile** | | | | | | | | | | | | | |
| Control | 0.7 (1.2) |  |  |  |  |  |  |  |  |  |  |  |  |
| AD | 1.5 (1.1) | 2.50 | 0.73, 8.60 | 0.367 |  |  |  |  |  |  |  |  |  |
| nfvPPA | 4.1 (1.5) | **8.31** | **2.53, 27.32** | **<0.001** | **3.32** | **1.70, 6.48** | **<0.001** |  |  |  |  |  |  |
| svPPA | 2.1 (1.5) | 3.19 | 0.94, 10.85 | 0.077 | 1.28 | 0.56, 2.92 | 1.00 | **0.38** | **0.18, 0.82** | **0.004** |  |  |  |
| bvFTD | 6.1 (2.0) | **14.83** | **4.78, 46.02** | **<0.001** | **5.92** | **3.25, 10.80** | **<0.001** | **1.78** | **1.07, 2.98** | **0.016** | **4.64** | **2.27, 9.48** | **<0.001** |
| **Mirthful misidentified as posed** | | | | | | | | | | | | | |
| Control | 0.9 (1.2) |  |  |  |  |  |  |  |  |  |  |  |  |
| AD | 1.0 (0.76) | 1.02 | 0.29, 3.64 | 1.00 |  |  |  |  |  |  |  |  |  |
| nfvPPA | 2.5 (1.5) | 2.88 | 0.86, 9.61 | 0.139 | **2.82** | **1.35, 5.91** | **0.001** |  |  |  |  |  |  |
| svPPA | 5.2 (1.6) | **8.11** | **2.97, 22.15** | **<0.001** | **7.94** | **3.52, 17.93** | **<0.001** | **2.82** | **1.39, 5.69** | **<0.001** |  |  |  |
| bvFTD | 1.5 (1.3) | 1.75 | 0.54, 5.64 | 1.00 | 1.71 | 0.76, 3.88 | 0.648 | 0.61 | 0.29, 1.27 | 0.589 | **0.22** | **0.10, 0.47** | **<0.001** |
| **Hostile misidentified as mirthful** | | | | | | | | | | | | | |
| Control | 1.4 (1.4) |  |  |  |  |  |  |  |  |  |  |  |  |
| AD | 2.9 (1.6) | 1.70 | 0.60, 4.80 | 1.00 |  |  |  |  |  |  |  |  |  |
| nfvPPA | 4.7 (2.1) | **3.41** | **1.20, 9.67** | **0.010** | **2.01** | **1.05, 3.84** | **0.026** |  |  |  |  |  |  |
| svPPA | 3.4 (1.3) | **2.72** | **1.29, 5.74** | **0.002** | 1.60 | 0.71, 3.61 | 1.00 | 0.80 | 0.35, 1.80 | 1.00 |  |  |  |
| bvFTD | 10.9 (1.9) | **19.5** | **8.63, 43.98** | **<0.001** | **11.47** | **6.02, 21.87** | **<0.001** | **5.71** | **3.00, 10.90** | **<0.001** | **7.15** | **3.93, 13.02** | **<0.001** |
| **Hostile misidentified as posed** | | | | | | | | | | | | | |
| Control | 1.2 (1.0) |  |  |  |  |  |  |  |  |  |  |  |  |
| AD | 2.9 (1.5) | 2.58 | 0.88, 7.64 | 0.138 |  |  |  |  |  |  |  |  |  |
| nfvPPA | 2.7 (2.8) | 2.43 | 0.57, 10.32 | 0.854 | 0.94 | 0.35, 2.51 | 1.00 |  |  |  |  |  |  |
| svPPA | 7.1 (1.7) | **10.35** | **5.50, 19.47** | **<0.001** | 4.00 | **1.60, 9.99** | **<0.001** | **4.26** | **1.15, 15.84** | **0.019** |  |  |  |
| bvFTD | 1.9 (1.4) | 1.59 | 0.68, 3.71 | 1.00 | 0.61 | 0.28, 1.34 | 0.777 | 0.65 | 0.21, 2.02 | 1.00 | **0.15** | **0.08, 0.31** | **<0.001** |
|  | | | | | | | | | | | | | |
|  |  |  |  |  |  |  |  |  |  |  |  |  |  |
| **Group** | **Error number (SD)** | **Control** | | | **AD** | | | **nfvPPA** | | | **svPPA** | | |
|  |  | OR | CI | P | OR | CI | P | OR | CI | P | OR | CI | P |
| **Posed misidentified as mirthful** | | | | | | | | | | | | | |
| Control | 1.5 (1.4) |  |  |  |  |  |  |  |  |  |  |  |  |
| AD | 3.0 (1.9) | 1.25 | 0.47, 3.31 | 1.00 |  |  |  |  |  |  |  |  |  |
| nfvPPA | 3.2 (1.5) | 1.54 | 0.62, 3.81 | 1.00 | 1.23 | 0.61, 2.48 | 1.00 |  |  |  |  |  |  |
| svPPA | 6.5 (1.7) | **6.29** | **2.95, 13.38** | **<0.001** | **5.04** | **2.41, 10.55** | **<0.001** | **4.09** | **2.20, 7.61** | **<0.001** |  |  |  |
| bvFTD | 3.9 (2.6) | 2.33 | 0.95, 5.71 | 0.078 | 1.87 | 0.93, 3.77 | 0.119 | 1.52 | 0.80, 2.89 | 0.693 | **0.37** | **0.19, 0.71** | **<0.001** |
| **Posed misidentified as hostile** | | | | | | | | | | | | | |
| Control | 1.1 (0.9) |  |  |  |  |  |  |  |  |  |  |  |  |
| AD | 2.3 (0.9) | **2.85** | **1.29, 6.30** | **0.002** |  |  |  |  |  |  |  |  |  |
| nfvPPA | 3.6 (1.5) | **4.70** | **2.05, 10.76** | **<0.001** | **1.65** | **1.06, 2.58** | **0.017** |  |  |  |  |  |  |
| svPPA | 2.8 (1.5) | **3.11** | **1.40, 6.89** | **0.001** | 1.09 | 0.58, 2.05 | 1.00 | 0.66 | 0.34, 1.28 | 0.800 |  |  |  |
| bvFTD | 4.9 (1.5) | **6.75** | **3.35, 13.57** | **<0.001** | **2.37** | **1.55, 3.62** | **<0.001** | 1.43 | 0.89, 2.32 | 0.345 | **2.17** | **1.24, 3.80** | **<0.001** |

Mean (standard deviation) numbers of each error type with pairwise comparisons expressed as adjusted odds ratios (with 95% confidence intervals). P values and CI are Bonferroni corrected for multiple pairwise comparisons. Statistically significant odds ratios (p_bonf_<0.05) are shown in bold. AD, patient group with typical Alzheimer’s disease; bvFTD, patient group with behavioural variant frontotemporal dementia; CI, 95% confidence intervals; Controls, healthy control group; Inverted, spectrally inverted laughter control condition; nfvPPA, patient group with nonfluent-agrammatic variant primary progressive aphasia; Numbers, spoken numbers control condition; SD, standard deviation; svPPA, patient group with semantic variant primary progressive aphasia.

**Supplementary Table S4.** Valence ratings of sound stimulus conditions and comparisons between participant groups

| **Condition** | **Group** | **Valence**  Mean (SD) | Controls | | AD | | nfvPPA | | svPPA | |
| --- | --- | --- | --- | --- | --- | --- | --- | --- | --- | --- |
|  |  |  | Difference (CI) | P | Difference (CI) | P | Difference (CI) | P | Difference (CI) | P |
| **Mirthful** | Controls | 4.13 (0.27) |  |  |  |  |  |  |  |  |
|  | AD | 4.01 (0.18) | -0.12 (-0.37, 0.13) | 1.00 |  |  |  |  |  |  |
|  | nfvPPA | **3.67 (0.18)** | **-0.44 (-0.69, -0.20)** | **<0.001** | **-0.32 (-0.52, -0.13)** | **<0.001** |  |  |  |  |
|  | svPPA | **3.21 (0.34)** | **-0.93 (-1.27, -0.59)** | **<0.001** | **-0.81 (-1.14, -0.47)** | **<0.001** | **-0.48 (-0.81, -0.15)** | **0.001** |  |  |
|  | bvFTD | **3.02 (0.38)** | **-1.11 (-1.41, -0.80)** | **<0.001** | **-0.99 (-1.27, -0.70)** | **<0.001** | **-0.66 (-0.94, -0.39)** | **<0.001** | -0.18 (-0.55, 0.19) | 1.00 |
| **Hostile** | Controls | 2.09 (0.24) |  |  |  |  |  |  |  |  |
|  | AD | 2.26 (0.19) | 0.18 (-0.05, 0.42) | 0.302 |  |  |  |  |  |  |
|  | nfvPPA | **2.44 (0.25)** | **0.37 (0.11, 0.64)** | **0.001** | 0.19 (-0.04, 0.43) | 0.190 |  |  |  |  |
|  | svPPA | **2.94 (0.19)** | **0.85 (0.64, 1.07)** | **<0.001** | **0.67 (0.43, 0.91)** | **<0.001** | **0.48 (0.21, 0.74)** | **<0.001** |  |  |
|  | bvFTD | **3.76 (0.44)** | **1.68 (1.39, 1.98)** | **<0.001** | **1.50 (1.17, 1.84)** | **<0.001** | **1.31 (0.97, 1.65)** | **<0.001** | **0.83 (0.54, 1.12)** | **<0.001** |
| **Posed** | Controls | 3.27 (0.17) |  |  |  |  |  |  |  |  |
|  | AD | 3.28 (0.09) | 0.02 (-0.16, 0.20) | 1.00 |  |  |  |  |  |  |
|  | nfvPPA | 3.25 (0.23) | -0.00 (-0.24, 0.24) | 1.00 | -0.02 (-0.22, 0.18) | 1.00 |  |  |  |  |
|  | svPPA | 3.18 (0.24) | -0.09 (-0.32, 0.14) | 1.00 | -0.11 (-0.36, 0.14) | 1.00 | -0.09 (-0.39, 0.21) | 1.00 |  |  |
|  | bvFTD | 3.11 (0.46) | -0.15 (-0.43, 0.13) | 1.00 | -0.17 (-0.49, 0.16) | 1.00 | -0.15 (-0.50, 0.20) | 1.00 | -0.06 (-0.39, 0.27) | 1.00 |
| **Inverted** | Controls | 1.41 (0.32) |  |  |  |  |  |  |  |  |
|  | AD | 1.53 (0.27) | 0.14 (-0.17, 0.44) | 1.00 |  |  |  |  |  |  |
|  | nfvPPA | 1.31 (0.27) | -0.08 (-0.39, 0.24) | 1.00 | -0.21 (-0.50, 0.08) | 0.360 |  |  |  |  |
|  | svPPA | 1.48 (0.42) | 0.07 (-0.33, 0.47) | 1.00 | -0.07 (-0.47, 0.33) | 1.00 | 0.15 (-0.26, 0.55) | 1.00 |  |  |
|  | bvFTD | 1.54 (0.47) | 0.14 (-0.20, 0.48) | 1.00 | 0.00 (-0.38, 0.38) | 1.00 | 0.22 (-0.15, 0.58) | 0.906 | 0.07 (-0.36, 0.50) | 1.00 |
| **Numbers** | Controls | 3.08 (0.11) |  |  |  |  |  |  |  |  |
|  | AD | 3.04 (0.06) | -0.03 (-0.19, 0.13) | 1.00 |  |  |  |  |  |  |
|  | nfvPPA | 3.08 (0.23) | 0.02 (-0.20, 0.25) | 1.00 | 0.05 (-0.15, 0.26) | 1.00 |  |  |  |  |
|  | svPPA | **3.68 (0.40)** | **0.60 (0.26, 0.94)** | **<0.001** | **0.63 (0.27, 1.00)** | **<0.001** | **0.58 (0.18, 0.97)** | **0.001** |  |  |
|  | bvFTD | 3.04 (0.25) | -0.03 (-0.22, 0.16) | 1.00 | 0.01 (-0.17, 0.18) | 1.00 | -0.05 (-0.29, .20) | 1.00 | **-0.63 (-1.00, -0.25)** | **<0.001** |

Mean (standard deviation) and adjusted differences between groups (with 95% confidence intervals) are shown for valence ratings of each sound condition. P values and CI are Bonferroni corrected for multiple pairwise comparisons. Statistically significant differences (p_bonf_<0.05) are shown in bold. AD, patient group with typical Alzheimer’s disease; bvFTD, patient group with behavioural variant frontotemporal dementia; CI, 95% confidence intervals; Controls, healthy control group; Inverted, spectrally inverted laughter control condition; nfvPPA, patient group with nonfluent-agrammatic variant primary progressive aphasia; Numbers, spoken numbers control condition; SD, standard deviation; svPPA, patient group with semantic variant primary progressive aphasia. progressive aphasia.

**Supplementary Table S5.** Acoustic parameters of laughter stimuli: comparisons between conditions

| **Laughter parameter** | **Mirthful** | **Hostile** | **Posed** | **Statistic** |
| --- | --- | --- | --- | --- |
| **Duration (s)** | 2.05 (0.15) | 2.02 (0.01) | 2.03 (0.07) | F=0.39, p=0.677 |
| **F0 (Hz)** | 558.12 (102.94)^a,b^ | 404.55 (121.16) | 500.06 (176.79) | F=8.66, p=0.0007 |
| **F0 range (Hz)** | 619.36 (122.02) ^a^ | 627.84 (174.32) ^a^ | 470.92 (142.76) | F=5.69, p=0.0062 |
| **Slope (Hz)** | 1910.09 (707.36) ^a^ | 1832.99 (701.37) ^a^ | 1117.74 (454.78) | F=7.64, p=0.0014 |
| **CoG (dB)** | 1396.24 (504.09) ^a^ | 1440.39 (323.46) ^a^ | 1021.84 (310.84)) | F=5.57, p=0.0069 |
| **HNR** | 8.81 (1.91) ^a^ | 9.53 (2.99) ^a^ | 2.45 (1.98) | F=44.29, p<0.001 |
| **F1** | 1022.78 (247.59) ^a^ | 1014.77 (94.90) ^a^ | 868.15 (86.62) | F=4.68, p=0.0143 |
| **F2** | 1764.92 (80.72) ^a,b^ | 1562.55 (102.41) | 1692.60 (56.52)^b^ | F=24.99, p<0.001 |
| **% unvoiced segments** | 47.70 (16.50) ^a^ | 56.87 (17.95) ^a^ | 32.16 (17.39) | F=8.35, p=0.0008 |

The table shows mean (standard deviation) data for key acoustic parameters of each laughter stimulus condition and statistics on comparisons between laughter conditions (see text for details). All parameter measures were derived from PRAAT©. ANOVAs, with post hoc Bonferroni-corrected paired t-tests where statistically significant main effects were found, were used to investigate parameter differences between laughter conditions. Statistically significant differences (post hoc p_bonf_<0.05) between laughter conditions on parameters of interest are coded as follows: ^a^greater than posed laughter; ^b^greater than hostile laughter. *F0* is the mean fundamental frequency (or pitch); *F0 range* is the difference between the lowest and highest fundamental frequency; *Slope* is the mean absolute F0 slope, which measures how sharply the pitch changes occur (difference between local F0 maximum and minimum / duration of corresponding pitch change); *Centre of gravity (CoG)* refers to the spectral centroid, which accounts for the weighting of noise across the sample and is an indicator of the timbre, or brightness, of a sound, with higher centres sounding brighter; *Harmonics-to-noise ratio (HNR)* is the average degree of periodicity in dB (a higher value indicates a purer, more tonal sound, and a lower value indicates a noisier, ‘breathier’ vocalisation); *F1 mean* and *F2 mean* are the first and second formants, peaks in the sound spectrum that help determine the identity of a vocalisation; *% unvoiced segments* refers to the proportion of sound segments that are unvoiced versus voiced – voiced segments are nearly periodic (harmonic), whilst unvoiced segments are nosier, and include exhalations and snorts.

**Supplementary Figure S1.** The relationship between laughter identification accuracy and daily life socio-emotional behaviour in patients with frontotemporal dementia syndromes.


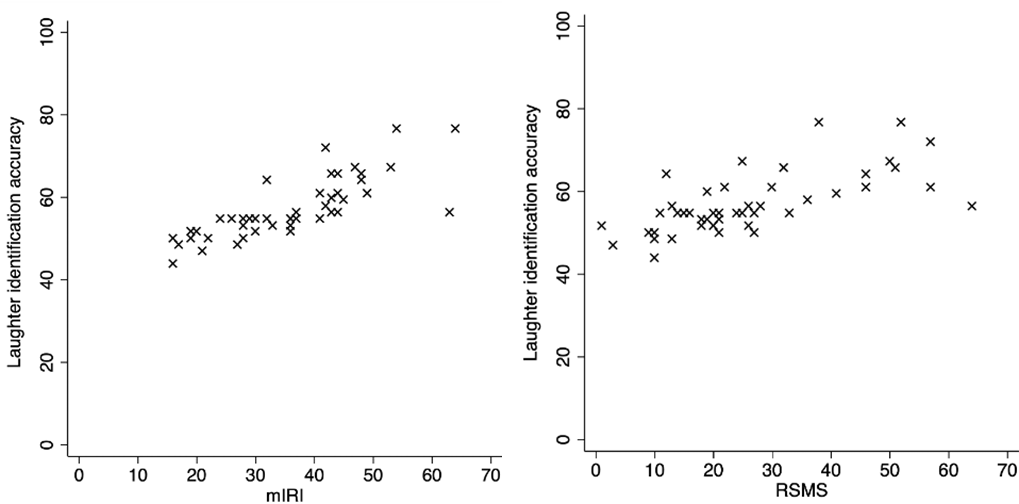


Scores on the Modified Interpersonal Reactivity Index (mIRI, left) and Revised Self-Monitoring Scale (RSMS, right) questionnaires (see main text for details) have been plotted against laughter identification accuracy (indexed as the percentage of all laughter trials correctly identified) for the combined frontotemporal dementia cohort. Crosses indicate individual data points.
